# Inside the Outbreaks: The Elite Medical Detectives of the Epidemic Intelligence Service

**DOI:** 10.3201/eid1607.100582

**Published:** 2010-07

**Authors:** Shira C. Shafir

**Affiliations:** Author affiliation: University of California Los Angeles School of Public Health, Los Angeles, California, USA

**Keywords:** EIS, epidemic intelligence service, review, Salmonella enteritidis, outbreaks, book review

In this book, Mark Pendergrast, a journalist by training, tries to present a comprehensive history of the Epidemic Intelligence Service (EIS). He successfully tells the stories of the elite group, but in attempting to cover so many of the outbreaks in which they have been involved, he loses the essence of their story.

Some of the outbreaks clearly have more detail than others. The story of the Cutter polio vaccine incident in 1955, the 1976 Ebola virus outbreak in Zaire, smallpox eradication, and the beginning of the AIDS epidemic in the United States are well documented. Unfortunately, even for these famous outbreaks, the stories are told better in other books singularly devoted to these topics rather than in this volume devoted to the role played by the EIS. Furthermore, most of the other outbreaks discussed in the book are covered in less than a page, and the author provides far too little detail. For example, the 1994 *Salmonella enteritidis* outbreak associated with Schwann’s ice cream that sickened >200,000 persons and was the largest common-source outbreak in the United States warranted only 2 paragraphs of coverage. The way in which Pendergrast describes the investigation—“He [Minnesota-based EIS officer Tom Hennessy] discovered that truck drivers were “back-hauling” liquid raw eggs on their return trip, and they were not cleaning their tankers sufficiently to prevent cross-contamination.”—makes the investigation seem simple and easy when it was, in fact, anything but simple and easy. Even though the individual outbreaks are well researched and written, the brevity with which each is portrayed makes light of the enormous effort involved in determining the source and controlling the outbreak. An outbreak investigation that may have taken weeks or months might be covered in the book in a mere 2 or 3 paragraphs, with little indication of how much the EIS officer(s) struggled to determine the etiologic agent and implement appropriate control measures.

If taken as a story of the history of the EIS, rather than as a story of the outbreaks themselves, the book achieves its primary goal. However, how interesting the history of the EIS is to persons who were not EIS officers or who do not aspire to be one is questionable. On the other hand, given that the book has a retail price of $28.00, and Pendergrast interviewed such an impressive number of EIS alumni that any public health professional is bound to recognize at least 1 name, even with its flaws, the book is still a fast and entertaining read.

